# Genomic surveillance unfolds the SARS-CoV-2 transmission and divergence dynamics in Bangladesh

**DOI:** 10.3389/fgene.2022.966939

**Published:** 2022-09-26

**Authors:** Tushar Ahmed Shishir, Taslimun Jannat, Iftekhar Bin Naser

**Affiliations:** ^1^ Department of Mathematics and Natural Sciences, BRAC University, Dhaka, Bangladesh; ^2^ Rangamati General Hospital, Chattogram, Bangladesh

**Keywords:** SARS-CoV-2, COVID-19, genetic diversity, molecular surveillance, evolution, pandemic

## Abstract

The highly pathogenic virus SARS-CoV-2 has shattered the healthcare system of the world causing the COVID-19 pandemic since first detected in Wuhan, China. Therefore, scrutinizing the genome structure and tracing the transmission of the virus has gained enormous interest in designing appropriate intervention strategies to control the pandemic. In this report, we examined 4,622 sequences from Bangladesh and found that they belonged to thirty-five major PANGO lineages, while Delta alone accounted for 39%, and 78% were from just four primary lineages. Our research has also shown Dhaka to be the hub of viral transmission and observed the virus spreading back and forth across the country at different times by building a transmission network. The analysis resulted in 7,659 unique mutations, with an average of 24.61 missense mutations per sequence. Moreover, our analysis of genetic diversity and mutation patterns revealed that eight genes were under negative selection pressure to purify deleterious mutations, while three genes were under positive selection pressure. Together with an ongoing genomic surveillance program, these data will contribute to a better understanding of SARS-CoV-2, as well as its evolution pattern and pandemic characteristics in Bangladesh.

## 1 Introduction

Originating in Wuhan, China, SARS-CoV-2 has spread across all the countries and territories, infecting 539.45 million and causing the death of 6.33 million people till 10^th^ June 2022, resulting in a global economic crisis, which is the third zoonotic virus after MERS-CoV and SARS-CoV in 2012 and 2002 respectively (Cui et al., 2019; Dong et al., 2020; Zhu et al., 2020). The novel virus belonging to the Betacoronavirus genus and Coronaviridae family is a positive-sense, single-stranded ∼30 kb long RNA virus. Its genome contains 38% GC content (Zhou et al., 2020), prefers pyrimidine-rich codons over purines (Kandeel et al., 2020) and is organized into 11 open reading frames expressing 12 proteins, including two polypeptides, four structural proteins and other accessory proteins (Naqvi et al., 2020). Phylogenetically, the virus shares 96% identity with the strain BatCoV RaTG13 of *Rhinolophus affinis*, and genome sequences along with epidemiological data suggest that SARS-CoV-2 is primarily transmitted from bats to humans (Cui et al., 2019; Andersen et al., 2020; Zhou et al., 2020). A complete genome sequence of the virus was deposited in GenBank on 5th January (NC_045512.2) (Wu et al., 2020), followed by the submission of 9.74 million complete sequences to GISAID by 25th March 2022 (Elbe and Buckland-Merrett, 2017).

Since the first case was confirmed in Bangladesh on 8th March 2020, there have been 1.953 million positive cases and 29,131 deaths reported until 10^th^ June 2022 (Islam et al., 2020). Having such a large population makes Bangladesh more vulnerable to viral transmission, and it is labelled as the second-most infected nation in the South Asian region (Worldometer, 2021), despite the government imposing lockdowns, social distancing rules and mask mandates to control the situation. Therefore, it is crucial to shed light on the transmission and evolution of the virus inside the country to reduce the fatality, where genomic data analyses and surveillance comes into play, which can deliver immense information. Child Health Research Foundation reported the first SARS-CoV-2 genome sequence from Bangladesh on 12th May 2020 (Saha et al., 2020), followed by 6,919 further sequences until 31^st^ May 2022 (Elbe and Buckland-Merrett, 2017).

To date, Bangladesh has been affected by three waves of COVID-19 with different variants of concern (VOC), including Alpha, Beta, Delta, and Omicron (Elbe and Buckland-Merrett, 2017). VOC is the name given to a SARS-CoV-2 variant that has mutations in the spike protein receptor-binding domain which increase the binding affinity within the RBD-hACE2 complex and increases viral transmission (Choi and Smith, 2021; Sanyaolu et al., 2021). Consequently, the mutations are essential for studying since they alter the antigenic potentials of the epitopes and consequently affect pathogenicity, infectivity, transmissibility, and the evasion of host immunity. SARS-CoV-2 encodes an exoribonuclease that proofreads the errors during viral RNA synthesis; therefore, it has a lower mutation rate than other RNA viruses, which aids in enhancing its ability to adapt to their environment (Ogando et al., 2020; Gribble et al., 2021). Nevertheless, the virus is accumulating mutations across its genome, leading to the emergence of different variants over time. These mutations are not evenly distributed; for example, some genes are more prone to mutations than others are. Moreover, cytosine to uracil substitution is more common in SARS-CoV-2, reforming the transition/transversion ratio, which is negatively correlated with evolutionary time (Duchêne et al., 2015). Additionally, a variable vaccination rate among the countries increases the risk of SARS-CoV-2 mutating into a strain that is resistant to current vaccines and therapies. Consequently, it is essential to continuously study the mutations of SARS-CoV-2 in order to develop further effective vaccines and therapies, improve pandemic response, and reduce the impact of the pandemic on healthcare and clinical processes in the country.

To the best of our knowledge, most of the previous studies in Bangladesh addressed lineages distribution, source determination, and potential mutations with only a few sequences from the early phase of the outbreak (Rahman et al., 2021; Shishir et al., 2021). Therefore, in this work, we comprehensively analyzed 4692 SARS-CoV-2 sequences isolated from Bangladesh until 31^st^ May 2022 to understand the distribution of variants and mutation accumulation trends over time. We have thoroughly studied the temporal and geographical distribution of different lineages inside Bangladesh and built the transmission network to trace their back and forth circulation. To better understand the evolutionary dynamics of SARS-CoV-2 in Bangladesh over the last 2 years, we examined the genetic diversity among strains, gene-wise mutation distribution, and selection pressures.

## 2 Methods and materials

### 2.1 Sequence retrieval and lineage determination

Using completeness and coverage filters on the sequences, all the SARS-CoV-2 genomes submitted from Bangladesh until 31^st^ May 2022 were retrieved from the Global Initiative on Sharing All Influenza Data (GISAID) database (www.gisaid.org) (Elbe and Buckland-Merrett, 2017). Prior to downstream analysis, all sequences were quality checked and sequences with more than 5% ambiguous characters were omitted. The sorted sequences were then classified by Phylogenetic Assignment of Named Global Outbreak LINeages (Pangolin) with COVID-19 Lineage Assigner (https://pangolin.cog-uk.io/) (O’Toole et al., 2021). Furthermore, we excluded lineages carrying less than ten sequences to address the important lineages. We analyzed and visualized the sequence lineage distribution in R within the country.

### 2.2 Transmission analysis

First, the selected sequences were aligned using the Mafft algorithm (Katoh et al., 2018), followed by the construction of a maximum likelihood phylogenetic tree using IQ-TREE (Minh et al., 2020)and calibrating the tree based on time with TreeTime (Sagulenko et al., 2018). Using the StrainHub tool (De Bernardi Schneider et al., 2020), we built the SARS-CoV-2 transmission network in Bangladesh from the reconstructed tree and metadata.

### 2.3 Mutation analysis

We have aligned each sequence with the reference sequence (NC_045512.2) (Wu et al., 2020) using the minimap2 algorithm (Li, 2018) and called the variants with Samtools (Li et al., 2009). Additionally, SNP-sites (Page et al., 2016), CovSeq (Simonetti et al., 2021) and an online server Coronapp (Mercatelli et al., 2021) were used to detect the mutations present in the sequences and the common mutations from these four sources were considered. Finally, SNPeff was used to predict the impact of the mutations (Cingolani et al., 2012).

### 2.4 Effects of mutation

First of all, we used TASSEL software (Bradbury et al., 2007) to determine the nucleotide diversity (π) using a 20 base-pair window at five base-pair steps. Then we calculated the direction of selection in the sequences to know if diversity moves away from neutrality and to understand the pattern of evolution using the SLAC algorithm (Kosakovsky Pond and Frost, 2005) in the HyPhy software package (Kosakovsky Pond et al., 2020). Moreover, FEL (Kosakovsky Pond and Frost, 2005), and FUBAR (Murrell et al., 2013) methods were used to identify specific sites experiencing diversifying or purifying selection. Linkage disequilibrium among mutations prevalent in 10% or more sequences were calculated using AutoVem (Xi et al., 2021) and presented by the R2 index using HaploView (Barrett et al., 2005). Then, along with determining the nucleotide substitution bias, the expected and observed transition, transversion events as well as their ratio were calculated by the method used by Matyášek R, Kovařík A (Matyášek and Kovařík, 2020).

## 3 Results

### 3.1 SARS-CoV-2 lineage dynamics

To understand the diversity and transmission of the virus, we have confined and analyzed sequences from all administrative divisions of Bangladesh. There were 6,919 sequences submitted in GISAID till 31^st^ May 2022, but many of them were incomplete and lacked quality. Therefore, we filtered the sequences based on their completeness, coverage, and gaps, resulting in 4,984 sequences for downstream analysis. In all, these sequences belonged to 93 PANGO lineages, although many of these lineages carried very few sequences. Hence, we further filtered the sequences and kept only the lineages containing at least ten sequences, resulting in 4,692 sequences from 35 lineages (Supplementary Material S1). Overall, in the beginning, the country had strains that belonged to the fewest number of PANGO lineages, but the scenario has changed over time (Supplementary Material S2). Selected sequences belonging to thirty-five different PANGO lineages provided us with invaluable insight regarding patterns of pandemic and viral spread (Supplementary Material S2). As an example, 78% of the sequences were grouped into four lineages, where Delta (B.1.617.2) and its three major sub-lineages (AY.X) combined made up the highest 39% of the total sequences, while 20 out of thirty-five lineages occupied only 9% of sequences. The top ten most prevalent lineages were found to be B.1.617.2 (23.00%), B.1.1.25 (20.38%), BA.2 (9.89%), B.1.351.3 (7.16%), AY.131 (6.14%), AY.122 (4.65%), AY.127 (2.73%), AY.4.4 (2.56%), BA.1 (2.47%), and B.1.1 (2.37%).

#### 3.1.1 Temporal distribution of major lineages

**FIGURE 1:**
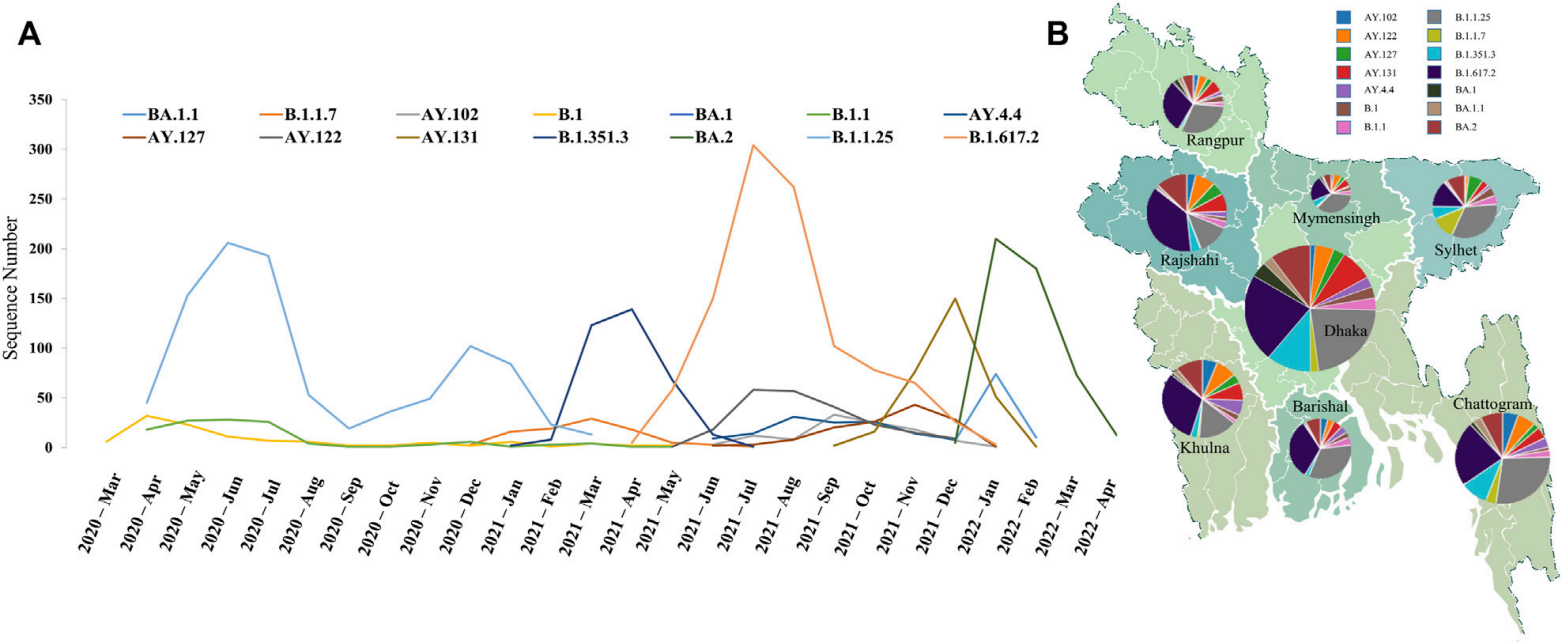
Distribution of major lineages in Bangladesh. **(A)** Geographic distribution of major lineages at administrative divisions. Dhaka contained the highest number of sequences and maximum diversity, while Mymensingh was the least diverse division. **(B)** Temporal distribution of major lineages. Maximum diversity was observed after the introduction of the Delta variant due to the emergence of different sub-lineage of it. Three major peaks depict the three variants responsible for three COVID-19 waves in Bangladesh.

We found that Bangladesh was infected by a large number of viruses from several lineages, with the highest diversification occurring between July and September of 2021 with sequences from 20 to 23 lineages (Supplementary Material S2). The early phase of the pandemic in Bangladesh was started by the introduction of lineage B.1 in March 2020. Multiple occurrences of the introduction of COVID-19 from different countries have previously been reported; for instance, Dhaka was first exposed to COVID-19 with strains from the United Kingdom, while Chattogram was exposed to strains from Saudi Arabia (Shishir et al., 2021). The early phase of the pandemic was generally dominated by strains imported from other countries, but as the pandemic progressed, mutations changed the dynamics and the linage B.1.1.25 took over, with B.1 gradually declining (Figure 1A). B.1.1.25 was the highest prevalent strain until January 2021. Later, the Beta variant (B.1.351) was reported in November 2020, followed by the Alpha variant (B.1.1.7) in December 2020. The B.1.1.7 lineage started taking over the B.1.1.25 lineage following its introduction. This linage was the most frequently detected variant in February 2021, while Beta variants were very less numerous. Despite this, a sub-lineage of Beta variants (B.1.351.3) emerged and outnumbered the Alpha variant in March 2021 (Supplementary Material S2). However, the dominance of B.1.351.3 did not last long due to the introduction of the deadly delta variant (B.1.617.2).

According to our analysis, B.1.617.2 was the most dominant strain within a month after its introduction in April 2021. A number of distinct A lineages have also been observed, which were mostly sub-lineages of the delta variant, possibly due to the increased transmissibility of the variant. Specifically, AY.122 increased significantly from September 2021 while B.1.617.2 was declining. Meanwhile, the AY.131 lineage first appeared in Bangladesh in October 2021 and surpassed all other variants in November 2021; more than half the sequences of December 2021 came from this lineage. This variant was eventually replaced by another highly transmissible variant named Omicron (B.1.1.529). The Omicron variant first emerged in Bangladesh in December 2021 and took over within a month, ultimately leading to the third wave of infections. Initially, the BA.1 sub-lineage of the Omicron variant dominated. However, BA.2 has gained a significant growth advantage over BA.1 and has taken over.

#### 3.1.2 Regional distribution of different lineages

**FIGURE 2:**
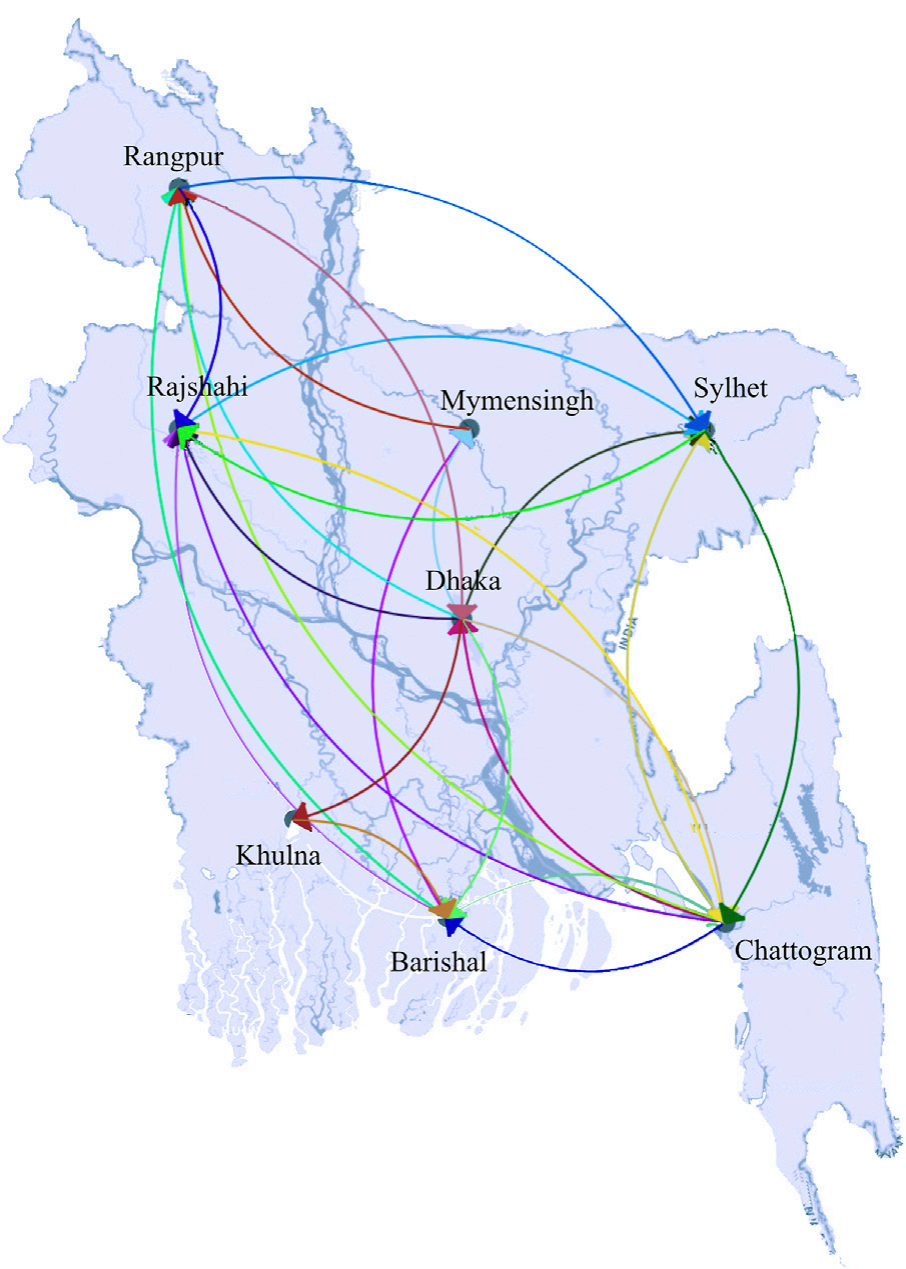
Transmission of SARS-CoV-2 in Bangladesh. Unlike others, Dhaka was connected with all other parts of the country, therefore recognized as a viral transmission hub. Arrows at the tip of the line dictate the direction of transmission.

We then conducted a geographical analysis in order to determine whether the variants were distributed evenly across Bangladesh’s administrative divisions. In terms of geographical distribution, Dhaka had the most diversified sequences from all thirty-five lineages, followed by Chattogram with 31. On the contrary, Mymensingh and Rangpur were less diverse areas with sequences from only 22 and 24 lineages, respectively, where most of the lineages represented only one or two sequences (Figure 1B). Initially, the Alpha variant was detected in Sylhet, and it has since spread to the other five divisions with the exception of Barishal and Rangpur, where the Delta and Omicron variants were first discovered in Dhaka. As a whole, the ratio of the dominant lineages was similar throughout the country, and our analyses of the transmission network indicate that Dhaka was the center of viral spread throughout the country (Figure 2). Area-specific detailed chronological distribution of SARS-CoV-2 variants is provided in the supplementary file (Supplementary Material S2).

To get a clearer idea of the viral circulation trend in different divisions of the country, we extensively analyzed the variants present there chronologically. We figured out that the whole country was mostly filled with a few major lineages throughout the time, but interestingly their dominance varied. We have seen that some lineages were missing from a particular area at a particular time and then reappeared, maybe due to mass people’s movement from other areas. For example, B.1.1 lineages were present in Mymensingh from the very beginning till June 2020. Then, this variant was missing there for 5 months but reappeared in the middle of December 2020. However, the variant was found present during this period in Dhaka and Chattogram. On the other hand, the sub-lineages of Beta variant B.1.351.3 were missing in Sylhet for 2 months from February to March 2021 and appeared again in April 2021, while was present in other divisions during this time. Several other back and forth circulation of strains were observed, for example, AY.100 and AY.102 in Dhaka. Detailed circulation of the variants information is provided in the supplementary file (Supplementary Material S2).

**FIGURE 3:**
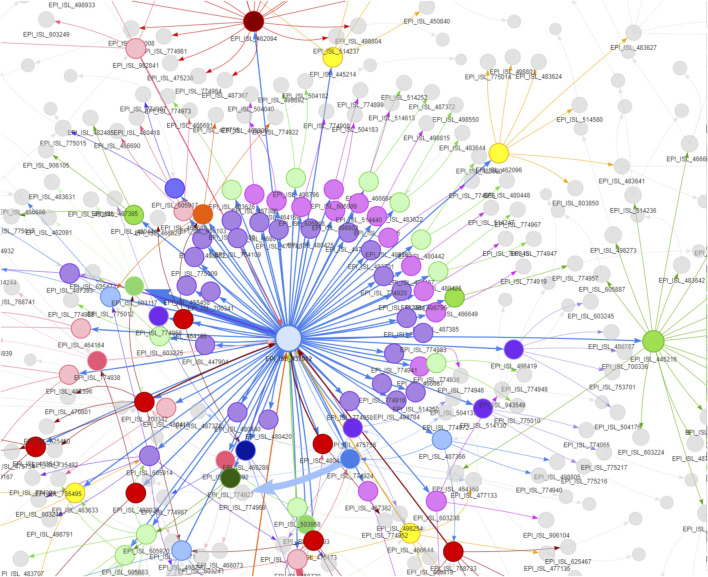
Strain to strain transmission network of the SARS-CoV-2 in Bangladesh. The sizes of nodes are proportional to the number of sequences a cluster contains and the thickness of the lines and arrows represent the frequency of transmission. The arrows reflect the direction of transmission among the viral clusters. The first sequence from the country is at the center of the network, and different clusters originated from the very first sequence, which gave rise to further subgroups; eventually, tips of the network reached.

Finally, we have built a viral transmission network using all our analysis data set sequences. Dhaka was found to be the center of viral transmission and directly connected with all other locations, while others were not. For example, we did not find any direct connection between Chattogram with Khulna and Mymensingh, Rangpur with Khulna, and Barisal did not have any connection with Sylhet (Figure 2). In addition, a strain-specific transmission network reveals the connections among different clusters and routes of viral spread from root to tip (Figure 3). With the time-calibrated analysis, we have observed that the sequences from Dhaka remain at the center of the network and determine the course of transmission forming connections with several subgroups.

### 3.2 Mutation analysis summary

**FIGURE 4:**
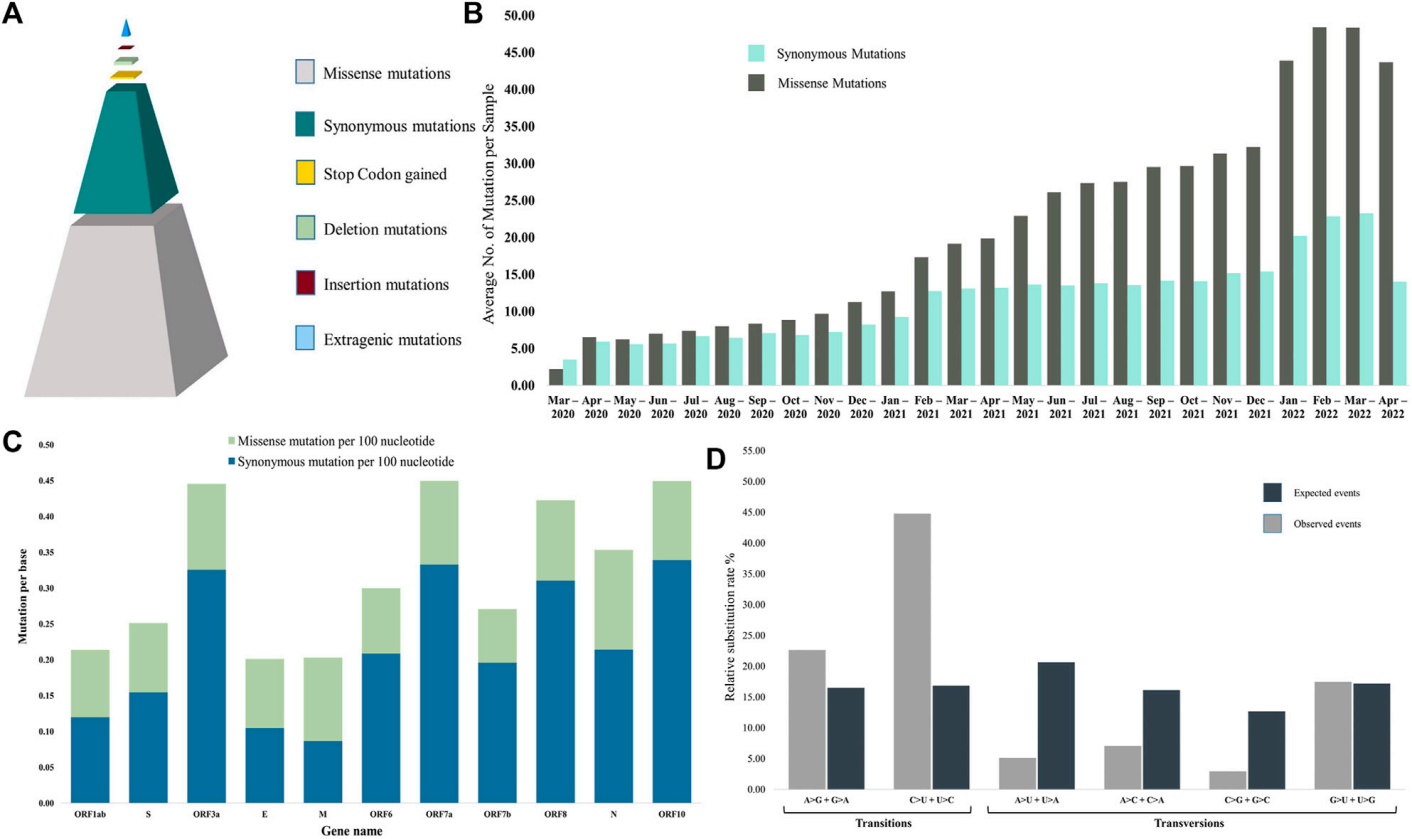
Summary of mutational events. **(A)** Type of mutations among the sequences. Considering all the unique mutations, missense mutations were found to be the most prevalent event. **(B)** The average number of mutations per sequence in each month. Although the number of mutations gradually increased with time, we observed a sharp increase from January 2021 when the Alpha variant entered the country. Moreover, more non-synonymous mutations emerged with time than synonymous mutations. **(C)** Percentage of mutation per base in each gene. ORF3a had the highest density of mutations, while Envelop protein is the least mutated. Missense mutations are more prevalent than synonymous mutations. **(D)** Nucleotide substitution rates for each of the four nucleotides among the SARS-CoV-2 genomes. Transition events were more prevalent than transversion events. C > U substitution rate was more than three times higher than the expected rate.

Up to the present study, we have found 7,659 unique mutations present in 4,692 sequences where 482 were extragenic mutations, and the rest were in the coding regions. In the coding region, a total of 4,103 missense, 2,865 synonymous, ten insertion, 125 deletion and 74 premature stop codon mutations were observed (Figure 4A). Moreover, our analysis demonstrated 37.64 mutations per sequence, where 24.61 mutations were missense, and the ratio of acquiring missense over synonymous mutations increased gradually (Figure 4B). We have seen the number of mutations increase gradually over time, yet nearly 29% of the sequences carried less than 30 mutations, and more than 55.25% of sequences had 30 to 50 mutations. The highest number of mutations detected was 78 in two strains isolated from Dhaka on 28^th^ February 2022, and the lowest number was only one found in a sequence from 11^th^ May 2021. Figure 4B clearly demonstrates two remarkable rises in mutations, one in February 2021 due to the introduction of Delta variants. Another sharp rise was observed in January 2022 because of the highly transmissible Omicron variant with a large number of mutations in the spike protein. However, the individual genes went through mutation distinctively. Therefore, we thoroughly carried out the mutational analysis of all the SARS-CoV-2 sequences from Bangladesh and summarized the results in Table 1 and Figure 4.

**TABLE 1:** SARS-CoV-2 mutation summary on individual genes.

ORF	No. of non-mutant sequences	Percentage of mutated sequences (%)	No. of synonymous mutations	No. of missense mutations	Percentage of missense mutations (%)	Mutation per base	No. of frequent mutations (n ≥ 10%)	No. of insertion mutation	No. of deletion mutation	No. of stop codon gained
ORF1ab	1	99.98	1997	2,550	56.08	0.214	29	2	33	20
S	2	99.96	370	590	61.46	0.251	31	4	40	11
ORF3a	873	81.11	99	270	73.17	0.446	4	2	5	1
E	3,395	26.55	22	24	52.17	0.202	1	0	0	1
M	1,423	69.21	78	58	42.65	0.203	3	0	1	2
ORF6	3,838	16.96	17	39	69.64	0.301	1	0	4	3
ORF7a	2,258	51.15	43	122	73.94	0.451	3	0	11	11
ORF7b	2,395	48.18	10	26	72.22	0.273	2	0	4	4
ORF8	1,604	65.30	41	114	73.55	0.424	1	1	11	13
N	78	98.31	175	270	60.67	0.353	12	1	15	3
ORF10	4,250	8.05	13	40	75.47	0.453	0	0	1	5

ORF10 and ORF7a harbored the highest mutation density with 0.453 and 0.451 mutations per base, respectively, although only 8.05% of sequences were found to carry mutations in ORF10. On the other hand, 99.98% and 99.96% of sequences had mutations in ORF1ab and S genes, but their mutation density was lower at 0.214 and 0.252, respectively. ORF6 was found to be the most stable gene of SARS-CoV-2 in sequences from Bangladesh, with only 16.96% sequences carrying mutations, 0.301 mutations per base and 69.64% missense mutations. ORF3a was identified to harbor the highest percentage (75.69%) of missense mutations. In comparison, the least percentage of missense mutations (42.65%) with 0.223 mutations per base was found in membrane protein-encoding gene M. It was clearly evident that non-structural proteins were subjected to more missense mutations than non-synonymous mutations compared with structural proteins (Figure 4C). In addition, we have found several deletions and insertion mutations where both the highest occurrences were found in the spike protein-coding S gene with 40 unique deletions and four insertions (Table 1). On the other hand, the highest number of unique stop codons were present in ORF1ab, with 40 out of 74 total stop codon mutations detected (Table 1).

Among the 7,786 mutations, 6,968 were SNP, where 4,697 and 2,271 were involved in transition and transversion events, respectively, rendering a transition transversion ratio of 2.07. Transition mutations were calculated to be more prevalent than expected if mutational events took place randomly, which clearly revealed the nucleotide substitution bias (Figure 4D). Then, transition mutation C > U was the most frequent event, being 30.67% of total mutations and 45.50% of transition mutations, followed by the transversion event G > U, which was 15.37% of the total mutations (Supplementary Material S3).

**FIGURE 5:**
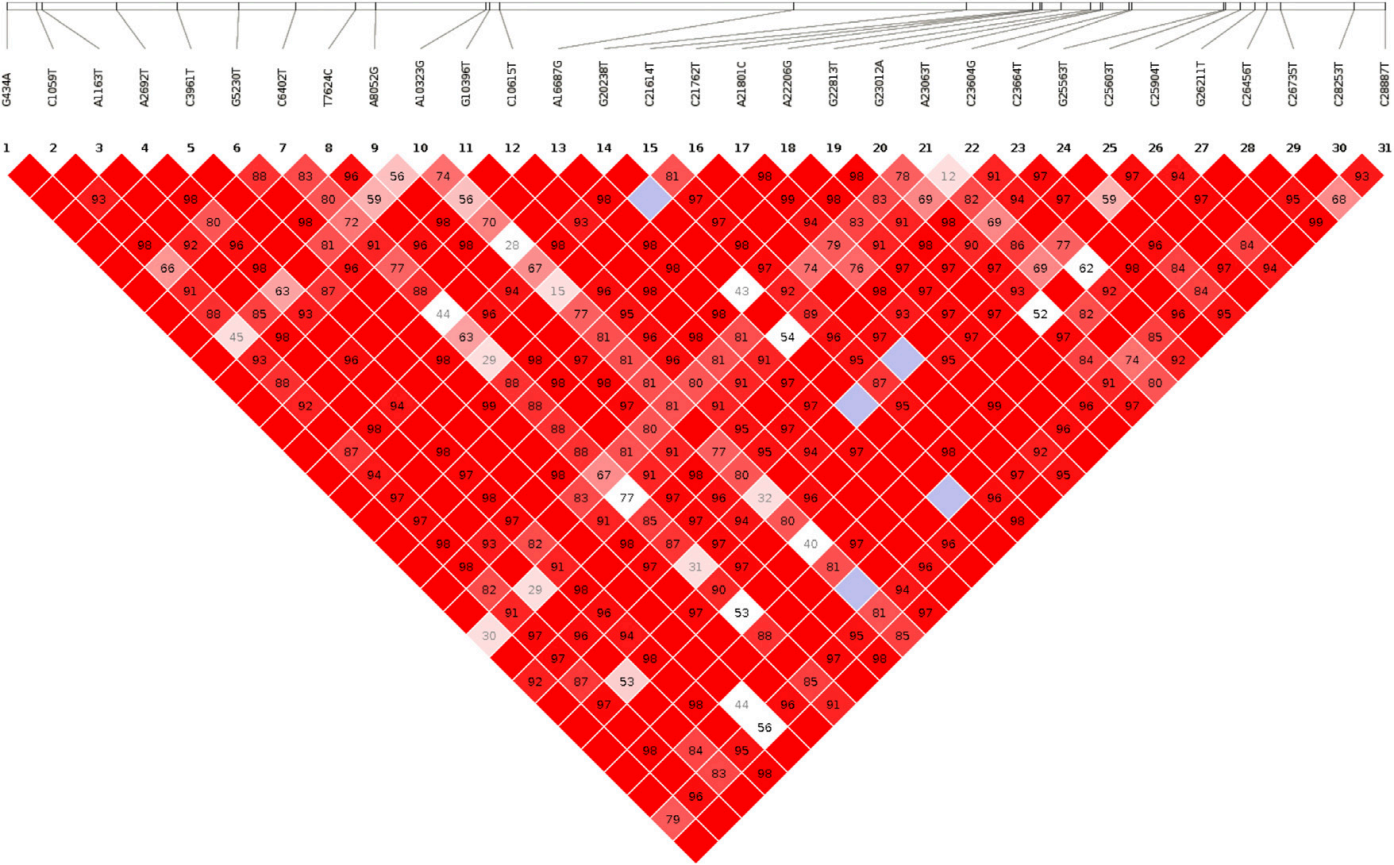
Linkage disequilibrium plot. The LD plot is generated considering the most prevalent SNPs. The number at the top denotes the SNP position, and squares are colored by standard (D'/LOD). The brighter red color indicates a higher D′ value and vice versa. The number in square is r^2^ value.

Then, out of the ten most prevalent mutations in Bangladesh, three were extragenic, one was synonymous, and six were missense mutations, where 23403A>G (missense mutation) was the highest prevalent, followed by the second highest 14408C>T (missense mutation) which resembles the global scenario and these two mutations appeared together with 3,037>C>T (synonymous mutation). Among the top seven mutations in the coding region, three were in the spike protein (D614G, P681R and T478K), two were in the ORF1ab (P4715L and F924F), one was in membrane protein (I82T), and another was in ORF3a (S26L). In addition, these seven mutations were highly prevalent throughout the world and were found in multiple lineages of the virus. Furthermore, 1163A>T (nsp2: I120F) was a highly prevalent and unique mutation found in Bangladesh from the beginning of the pandemic, whereas it was absent from other countries during that period. There were more than 21% of sequences containing this mutation. Then, from the linkage disequilibrium analysis (LD), we found that all the mutations mentioned had a perfect correlation (R2 = 1.00) and have been present together since their occurrence (Figure 5). According to our sequence analysis, the 1163A>T mutation had perfect LD (R2 = 1.00) with 20 additional mutations, considering the high frequency of mutations present in at least 10% of the sequences. Additionally, several other mutations were also observed to occur concurrently with very high LD values (R2 > 0.90) (Figure 5).

### 3.3 Effect of the mutations

The mutations affect viral infectivity, transmissibility, virulence, viral fitness, selection pressure, proteome structure, and evolution. Overall, the SARS-CoV-2 genomes had very high nucleotide identity with an average of 37.64 mutations and low overall nucleotide diversity (π) 0.004. Although overall nucleotide diversity was lower, it varied from gene to gene. For example, ORF8 had the highest nucleotide diversity (0.01543), while gene ORF10 was most stable with a π value of 0.00059 (Table 2). Analyzing the Bangladeshi sequences, the most diverse spot of the genome was in the spike protein gene at position 23009 with nucleotide diversity value π = 0.16372 while the least diverse spot found was at position 11069 of ORF1ab with a π value of 0.00005.

**TABLE 2:** Summary of the mutational effects on each protein.

ORF	Nucleotide diversity (π)	dN/dS	No. of sites under positive selection	No. of sites under negative selection
ORF1ab	0.0017	0.579	28	103
S	0.00526	0.74	7	48
ORF3a	0.00292	1.479	2	11
E	0.00223	0.479	0	2
M	0.00206	0.371	1	6
ORF6	0.00452	0.928	5	18
ORF7a	0.00549	.987	0	3
ORF7b	0.0046	1.44	0	0
ORF8	0.01543	0.941	1	9
N	0.00579	0.841	3	14
ORF10	0.00059	1.17	0	2

(dN/dS > 1 is positive selection, dN/dS = 1 neutral selection, dN/dS < 1 negative selection, dN/dS = 0 is conserved region).

It is also important to note that nucleotide diversity is heavily influenced by natural selection within populations. Therefore, we have analyzed the natural selection pattern of SARS-CoV-2 using several evolutionary algorithms. As we have observed, most of the genes overall had lower nucleotide diversity than other human viruses such as H1N1, H3N2, parainfuenza viruses (Martinez-Hernandez et al., 2010; Beck et al., 2012; López-Labrador et al., 2016), which is consistent with purifying selection. Using the phylogenetically corrected SLAC method with a default *p*-value of 0.1, we calculated the dN/dS and found that eight of the eleven genes were under negative selection pressure, which signifies the low nucleotide diversity. Additionally, three genes (ORF3a, ORF7b, and ORF10) were under positive selection pressure or directional selection since the mutations present in them were advantageous to them, as a result, their frequencies were increasing. On the other hand, rest of the genes were experiencing negative evolution pressure to eliminate the deleterious mutations that they have acquired from random mutations. It is likely that eighty-two percent of the 7,659 unique mutations were present in sequences below ten, possibly due to their deleterious effects on the virus, and that these mutations were gradually purged by the purifying selection pressure. This higher number of mutations with low frequency is also indicative of a demographic process known as population expansion, which might have resulted in a reduction in the overall genetic diversity. Following that, we thoroughly analyzed the specific sites under selection pressure using the FEL, and FUBAR methods. There were only 47 sites that were under positive or divergent selection pressure compared to 190 sites that were under negative or purifying selection (Supplementary Material S3). Negative selection pressure was found in ORF1ab, S, ORF3a, M, ORF6, ORF8, and N genes, while positive selection pressure was found in all genes except ORF7b.

**FIGURE 6:**
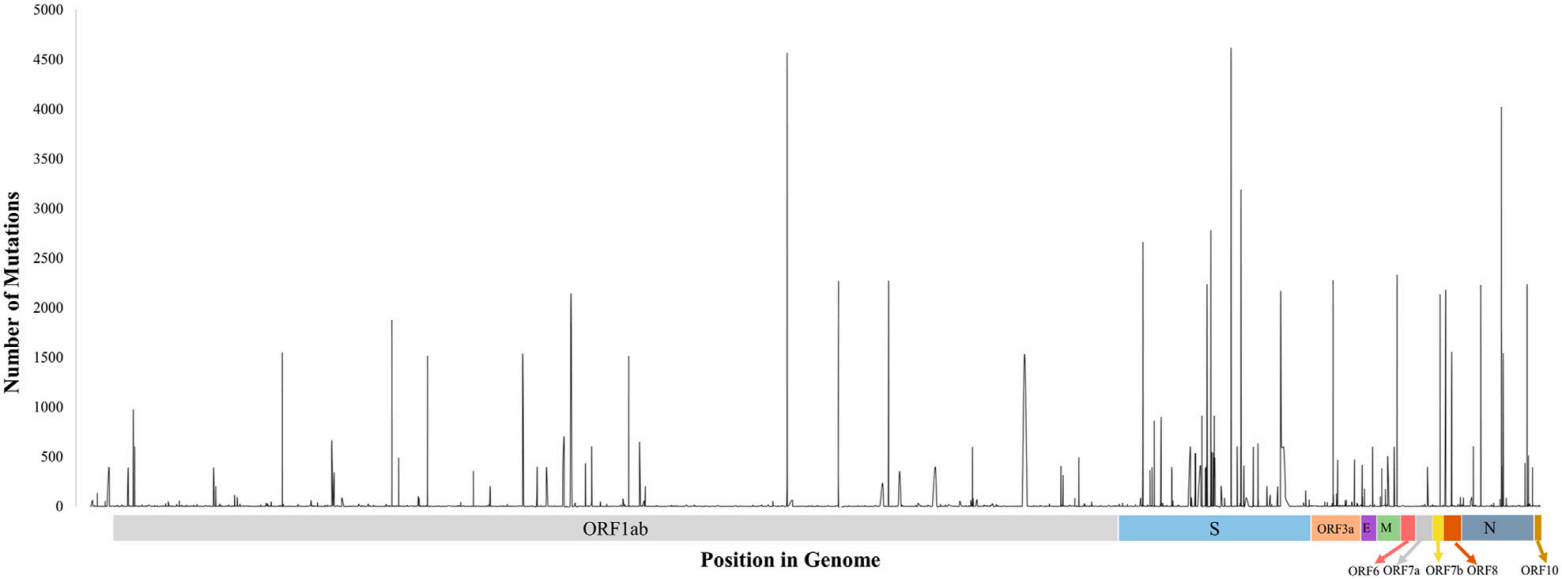
Distribution of missense mutations in the genome. The height of the spikes is proportional to the number of sequences that got mutation at that location. Regions between these spikes are stable, which could be targeted for further vaccine and therapeutics development.

Finally, these mutations affected the virus from the evolutionary perspective and shook the stability of the proteins they encode. Most of the mutations were previously reported to affect the stability of the whole proteome of SARS-CoV-2 negatively. However, all the genes were not affected to the same extent by mutational events (Figure 6). For example, only 42.65% of mutations on the membrane protein-coding M gene were missense which was 73.55% in the case of ORF3a (Table 1). As of now, vaccines and therapies target the spike protein, which is highly mutated. That is one the reasons why people continue to develop symptoms after successful vaccination. It is possible that current vaccines and therapies will not work in future due to a high number of mutations occurring. The less affected genes could therefore be targeted for medicine and vaccine development. Figure 6 shows spikes that represent mutations, and the height of the spikes is proportional to the number of mutations that have taken place at that position in the genome. As we can see, there are plenty of stable regions between the spikes, which could be targeted for therapeutics and vaccine development against SARS-CoV-2.

## 4 Discussion

SARS-CoV-2 has been circulating in Bangladesh for over 2 years, and many strains are sequenced from different parts of the country, helping us carry out analysis to depict different variants, transmission, and evolution inside the country. Investigating 4,692 whole-genome sequences from Bangladesh, we have seen B.1.1.25 lineage was dominant since the beginning, but since March 2021, another lineage beta variant (B.1.351.3), was dominant. However, In April 2021 Delta variant emerged and dominated other variants until the arrival of Omicron. Omicron variants BA.1 and BA.2 are found in Bangladesh, and currently, BA.2 is the dominant variant., 20 distinct mutations in the spike protein differentiate the two sub-lineages, and BA.2 displays a marked decreased sensitivity to many neutralizing monoclonal antibodies (mAbs) when compared to previous VOCs (Deen et al., 2020). Therefore, with further mutations, this BA.2 sub-lineage is keeping the risk of having another COVID-19 wave alive in the country.

On the other hand, geographical analysis depicts Dhaka and Chattogram containing a more diversified number of sequences than other parts of the country. Our analysis has limitations at this point because we had a higher number of sequences from these two areas than others. The sequences were more diversified in the first phase of the pandemic. However, with the arrival of the Delta and Omicron variants, the divergence reduced drastically, maybe due to viral adaptation following the “Survival of the fittest” theory of natural selection. Additionally, we have seen Dhaka being the viral transmission hub, which is obvious since it is the capital city of Bangladesh. From extensive analysis, we have built the SARS-CoV-2 transmission network between different administrative divisions and observed the back-and-forth circulation of the virus inside the country. This situation arose due to a lack of restrictions on mass movement; public gatherings and other socioeconomic events.

From the mutational perspective, we have seen 37.64 mutations per sample where on average 24.61were coding variants, which happens to be significantly higher than the global average of 7.23, reported in July 2020 (Mercatelli and Giorgi, 2020). This sharp rise of mutations indicates the SARS-CoV-2 might be facing strong challenges from the host’s immunologic response in addition to random regular mutational events of RNA viruses. At the nucleotide level, 67.41% of the mutations tend to mutate hydrophilic amino acids into hydrophobic ones (Matyášek and Kovařík, 2020) and are involved in altering the proline, which is known to be a strong helix breaker (Li et al., 1996). Therefore, proline to another amino acid shift might have a deleterious effect on the SARS-CoV-2 proteome.

Moreover, we have observed that most of the genes were under negative selection pressure while only three non-structural protein-coding genes were under Darwinian (positive) selection, indicating that most of the random mutations were deleterious for SARS-CoV-2 (Lin et al., 2019), which could be attributed to the immunologic potential of people in Bangladesh and our demography. However, the dN/dS ratio of the receptor-binding domain (RBD) of the spike protein was higher, suggesting that mutations in this region were advantageous. The result correlates with the emergence of different variants of concerns like Alpha, Beta, Delta, and Omicron. The RBD region is considered the most important part of the virus since it attaches to ACE2 during viral infection to host cells. It is possible that these advantageous mutations may increase pathogenicity, infectivity, transmissibility, and enable it to evade host immunity (Lan et al., 2020; Barros et al., 2021; Xu et al., 2021). Furthermore, most of the current therapies and vaccines target the interaction between BRD and ACE2. Thus, a higher dN/dS ratio may also signal the emergence of new deadly variants in the future with further mutations in this region and the failure of vaccines. In spite of the fact that we used several algorithms to detect the natural selection pattern of SARS-CoV-2 in Bangladesh, our analysis has some limitations, including sequencing errors and artefacts resulting from laboratory recombination of the sequences. A further limitation is that we do not know the exact arrival time of the SARS-CoV-2 in Bangladesh. Therefore, we do not know if any important changes have occurred in the genome before the first virus was sequenced on 8 April 2020. In addition, only a small number of viruses were sequenced during these 2 years, and there were differences in sequencing symmetry among different regions of the country. Additionally, the algorithms used are not error-free, so it is possible that some of the results obtained are false positives. In light of all these factors, we are only able to provide a prediction of the evolutionary pattern of the virus, rather than a conclusive analysis.

To sum up, considering the limitations regarding sequence number variations in different parts of the country, we have thoroughly studied the virus circulation trend and analyzed all the mutations present, which are comprehensively reported in the supplementary files. This data would further facilitate researchers from various perspectives like investigating viral transmission, the connection among isolates, evolution patterns, and dynamics of divergence of the virus.

## Data Availability

The datasets presented in this study can be found in online repositories. The names of the repository/repositories and accession number(s) can be found below: https://doi.org/10.6084/m9.figshare.19608885.

## References

[B1] Andersen K. G. Rambaut A. Lipkin W. I. Holmes E. C. Garry R. F. (2020). The proximal origin of SARS-CoV-2. Nat. Med. 26, 450–452. 10.1038/s41591-020-0820-9 32284615 PMC7095063

[B2] Barrett J. C. Fry B. Maller J. Daly M. J. (2005). Haploview: Analysis and visualization of LD and haplotype maps. Bioinformatics 21, 263–265. 10.1093/bioinformatics/bth457 15297300

[B3] Barros E. P. Casalino L. Gaieb Z. Dommer A. C. Wang Y. Fallon L. (2021). The flexibility of ACE2 in the context of SARS-CoV-2 infection. Biophys. J. 120, 1072–1084. 10.1016/j.bpj.2020.10.036 33189680 PMC7661960

[B4] Beck E. T. He J. Nelson M. I. Bose M. E. Fan J. Kumar S. (2012). Genome sequencing and phylogenetic analysis of 39 human parainfluenza virus Type 1 strains isolated from 1997-2010. PLoS One 7, e46048. 10.1371/journal.pone.0046048 23029382 PMC3459887

[B5] Bradbury P. J. Zhang Z. Kroon D. E. Casstevens T. M. Ramdoss Y. Buckler E. S. (2007). Tassel: Software for association mapping of complex traits in diverse samples. Bioinformatics 23, 2633–2635. 10.1093/bioinformatics/btm308 17586829

[B6] Choi J. Y. Smith D. M. (2021). SARS-CoV-2 variants of concern. Yonsei Med. J. 62, 961–968. 10.3349/ymj.2021.62.11.961 34672129 PMC8542474

[B7] Cingolani P. Platts A. Wang L. L. Coon M. Nguyen T. Wang L. (2012). A program for annotating and predicting the effects of single nucleotide polymorphisms, SnpEff: SNPs in the genome of *Drosophila melanogaster* strain w1118; iso-2; iso-3. Fly. (Austin) 6, 80–92. 10.4161/fly.19695 22728672 PMC3679285

[B8] Cui J. Li F. Shi Z. L. (2019). Origin and evolution of pathogenic coronaviruses. Nat. Rev. Microbiol. 17, 181–192. 10.1038/s41579-018-0118-9 30531947 PMC7097006

[B9] De Bernardi Schneider A. Ford C. T. Hostager R. Williams J. Cioce M. Çatalyürek Ü. V. (2020). StrainHub: A phylogenetic tool to construct pathogen transmission networks. Bioinformatics 36, 945–947. 10.1093/bioinformatics/btz646 31418766 PMC8215912

[B10] Deen J. Mengel M. A. Clemens J. D. (2020). Epidemiology of cholera. Vaccine 38, A31–A40. 10.1016/j.vaccine.2019.07.078 31395455

[B11] Dong E. Du H. Gardner L. (2020). An interactive web-based dashboard to track COVID-19 in real time. Lancet. Infect. Dis. 20, 533–534. 10.1016/S1473-3099(20)30120-1 32087114 PMC7159018

[B12] Duchêne S. Ho S. Y. Holmes E. C. (2015). Declining transition/transversion ratios through time reveal limitations to the accuracy of nucleotide substitution models. BMC Evol. Biol. 15, 36. 10.1186/s12862-015-0312-6 25886870 PMC4358783

[B13] Elbe S. Buckland-Merrett G. (2017). Data, disease and diplomacy: GISAID’s innovative contribution to global health. Glob. Chall. 1, 33–46. 10.1002/gch2.1018 31565258 PMC6607375

[B14] Gribble J. Stevens L. J. Agostini M. L. Anderson-Daniels J. Chappell J. D. Lu X. (2021). The coronavirus proofreading exoribonuclease mediates extensive viral recombination. PLoS Pathog. 17, e1009226. 10.1371/journal.ppat.1009226 33465137 PMC7846108

[B15] Islam M. T. Talukder A. K. Siddiqui M. N. Islam T. (2020). Tackling the COVID-19 pandemic: The Bangladesh perspective. J. Public health Res. 9, 389–397. 10.4081/jphr.2020.1794 PMC7582102 33117758

[B16] Kandeel M. Ibrahim A. Fayez M. Al-Nazawi M. (2020). From SARS and MERS CoVs to SARS-CoV-2: Moving toward more biased codon usage in viral structural and nonstructural genes. J. Med. Virol. 92, 660–666. 10.1002/jmv.25754 32159237 PMC7228358

[B17] Katoh K. Rozewicki J. Yamada K. D. (2018). MAFFT online service: Multiple sequence alignment, interactive sequence choice and visualization. Brief. Bioinform. 20, 1160–1166. 10.1093/bib/bbx108 PMC6781576 28968734

[B18] Kosakovsky Pond S. L. Frost S. D. W. (2005). Not so different after all: A comparison of methods for detecting amino acid sites under selection. Mol. Biol. Evol. 22, 1208–1222. 10.1093/molbev/msi105 15703242

[B19] Kosakovsky Pond S. L. Poon A. F. Y. Velazquez R. Weaver S. Hepler N. L. Murrell B. (2020). HyPhy 2.5 - a customizable platform for evolutionary hypothesis testing using phylogenies. Mol. Biol. Evol. 37, 295–299. 10.1093/molbev/msz197 31504749 PMC8204705

[B20] Lan J. Ge J. Yu J. Shan S. Zhou H. Fan S. (2020). Structure of the SARS-CoV-2 spike receptor-binding domain bound to the ACE2 receptor. Nature 581, 215–220. 10.1038/s41586-020-2180-5 32225176

[B21] Li H. Handsaker B. Wysoker A. Fennell T. Ruan J. Homer N. (2009). The sequence alignment/map format and SAMtools. Bioinformatics 25, 2078–2079. 10.1093/bioinformatics/btp352 19505943 PMC2723002

[B22] Li H. (2018). Minimap2: Pairwise alignment for nucleotide sequences. Bioinformatics 34, 3094–3100. 10.1093/bioinformatics/bty191 29750242 PMC6137996

[B23] Li S. C. Goto N. K. Williams K. A. Deber C. M. (1996). Alpha-helical, but not beta-sheet, propensity of proline is determined by peptide environment. Proc. Natl. Acad. Sci. U. S. A. 93, 6676–6681. 10.1073/pnas.93.13.6676 8692877 PMC39085

[B24] Lin J. J. Bhattacharjee M. J. Yu C. P. Tseng Y. Y. Li W. H. (2019). Many human RNA viruses show extraordinarily stringent selective constraints on protein evolution. Proc. Natl. Acad. Sci. U. S. A. 116, 19009–19018. 10.1073/pnas.1907626116 31484772 PMC6754614

[B25] López-Labrador F. X. Natividad-Sancho A. Pisareva M. Komissarov A. Salvatierra K. Fadeev A. (2016). Genetic characterization of influenza viruses from influenza-related hospital admissions in the St. Petersburg and Valencia sites of the Global Influenza Hospital Surveillance Network during the 2013/14 influenza season. J. Clin. Virol. 84, 32–38. 10.1016/j.jcv.2016.09.006 27690141

[B26] Martinez-Hernandez F. Jimenez-Gonzalez D. E. Martinez-Flores A. Villalobos-Castillejos G. Vaughan G. Kawa-Karasik S. (2010). What happened after the initial global spread of pandemic human influenza virus A (H1N1)? A population genetics approach. Virol. J. 7, 196. 10.1186/1743-422X-7-196 20727188 PMC2936310

[B27] Matyášek R. Kovařík A. (2020). Mutation patterns of human SARS-CoV-2 and bat RATG13 coronavirus genomes are strongly biased towards C>U transitions, indicating rapid evolution in their hosts. Genes 11, 761. 10.3390/genes11070761 PMC7397057 32646049

[B28] Mercatelli D. Giorgi F. M. (2020). Geographic and genomic distribution of SARS-CoV-2 mutations. Front. Microbiol. 11, 1800. 10.3389/fmicb.2020.01800 32793182 PMC7387429

[B29] Mercatelli D. Triboli L. Fornasari E. Ray F. Giorgi F. M. (2021). Coronapp: A web application to annotate and monitor SARS-CoV-2 mutations. J. Med. Virol. 93, 3238–3245. 10.1002/jmv.26678 33205830 PMC7753722

[B30] Minh B. Q. Schmidt H. A. Chernomor O. Schrempf D. Woodhams M. D. Von Haeseler A. (2020). IQ-TREE 2: New models and efficient methods for phylogenetic inference in the genomic era. Mol. Biol. Evol. 37, 1530–1534. 10.1093/molbev/msaa015 32011700 PMC7182206

[B31] Murrell B. Moola S. Mabona A. Weighill T. Sheward D. Kosakovsky Pond S. L. (2013). Fubar: A fast, unconstrained bayesian AppRoximation for inferring selection. Mol. Biol. Evol. 30, 1196–1205. 10.1093/molbev/mst030 23420840 PMC3670733

[B32] Naqvi A. A. T. Fatima K. Mohammad T. Fatima U. Singh I. K. Singh A. (2020). Insights into SARS-CoV-2 genome, structure, evolution, pathogenesis and therapies: Structural genomics approach. Biochim. Biophys. Acta. Mol. Basis Dis. 1866, 165878. 10.1016/j.bbadis.2020.165878 32544429 PMC7293463

[B33] Ogando N. S. Zevenhoven-Dobbe J. C. van der Meer Y. Bredenbeek P. J. Posthuma C. C. Snijder E. J. (2020). The enzymatic activity of the nsp14 exoribonuclease is critical for replication of MERS-CoV and SARS-CoV-2. J. Virol. 94, e01246-20. 10.1128/jvi.01246-20 PMC7654266 32938769

[B34] O’Toole Á. Scher E. Underwood A. Jackson B. Hill V. McCrone J. T. (2021). Assignment of epidemiological lineages in an emerging pandemic using the pangolin tool. Virus Evol. 7, veab064. 10.1093/ve/veab064 34527285 PMC8344591

[B35] Page A. J. Taylor B. Delaney A. J. Soares J. Seemann T. Keane J. A. (2016). SNP-Sites: Rapid efficient extraction of SNPs from multi-FASTA alignments. Microb. Genom. 2, e000056. 10.1099/mgen.0.000056 28348851 PMC5320690

[B36] Rahman M. M. Kader S. B. Rizvi S. M. S. (2021). Molecular characterization of SARS-CoV-2 from Bangladesh: Implications in genetic diversity, possible origin of the virus, and functional significance of the mutations. Heliyon 7, e07866. 10.1016/j.heliyon.2021.e07866 34458642 PMC8380069

[B37] Sagulenko P. Puller V. Neher R. A. (2018). TreeTime: Maximum-likelihood phylodynamic analysis. Virus Evol. 4, vex042. 10.1093/ve/vex042 29340210 PMC5758920

[B38] Saha S. Malaker R. Sajib M. S. I. Hasanuzzaman M. Rahman H. Ahmed Z. B. (2020). Complete genome sequence of a novel coronavirus (SARS-CoV-2) isolate from Bangladesh. Microbiol. Resour. Announc. 9, e00568-20. 10.1128/mra.00568-20 32527780 PMC7291105

[B39] Sanyaolu A. Okorie C. Marinkovic A. Haider N. Abbasi A. F. Jaferi U. (2021). The emerging SARS-CoV-2 variants of concern. Ther. Adv. Infect. Dis. 8, 20499361211024372. 10.1177/20499361211024372 34211709 PMC8216402

[B40] Shishir T. A. Naser I. Bin Faruque S. M. (2021). *In silico* comparative genomics of SARS-CoV-2 to determine the source and diversity of the pathogen in Bangladesh. PLoS One 16, e0245584. 10.1371/journal.pone.0245584 33471859 PMC7817022

[B41] Simonetti M. Zhang N. Harbers L. Milia M. G. Brossa S. Huong Nguyen T. T. (2021). COVseq is a cost-effective workflow for mass-scale SARS-CoV-2 genomic surveillance. Nat. Commun. 12, 3903. 10.1038/s41467-021-24078-9 34162869 PMC8222401

[B42] Worldometer (2021). COVID live update: 239, 169, 612 cases and 4, 875, 781 deaths from the coronavirus. Washington, DC: Worldometer (worldometers.info). Available at: https://www.worldometers.info/coronavirus/ (Accessed June 10, 2022).

[B43] Wu F. Zhao S. Yu B. Chen Y. M. Wang W. Song Z. G. (2020). A new coronavirus associated with human respiratory disease in China. Nature 579, 265–269. 10.1038/s41586-020-2008-3 32015508 PMC7094943

[B44] Xi B. Jiang D. Li S. Lon J. R. Bai Y. Lin S. (2021). AutoVEM: An automated tool to real-time monitor epidemic trends and key mutations in SARS-CoV-2 evolution. Comput. Struct. Biotechnol. J. 19, 1976–1985. 10.1016/j.csbj.2021.04.002 33841748 PMC8020629

[B45] Xu C. Wang Y. Liu C. Zhang C. Han W. Hong X. (2021). Conformational dynamics of SARS-CoV-2 trimeric spike glycoprotein in complex with receptor ACE2 revealed by cryo-EM. Sci. Adv. 7, eabe5575. 10.1126/sciadv.abe5575 33277323 PMC7775788

[B46] Zhou P. Yang X. L. Wang X. G. Hu B. Zhang L. Zhang W. (2020). A pneumonia outbreak associated with a new coronavirus of probable bat origin. Nature 579, 270–273. 10.1038/s41586-020-2012-7 32015507 PMC7095418

[B47] Zhu N. Zhang D. Wang W. Li X. Yang B. Song J. (2020). A novel coronavirus from patients with pneumonia in China, 2019. N. Engl. J. Med. 382, 727–733. 10.1056/nejmoa2001017 31978945 PMC7092803

