# Supplementary figures and images for: Genomic surveillance unfolds the SARS-CoV-2 transmission and divergence dynamics in Bangladesh
Source: Front Genet. 2022 Sep 26;13:966939. doi: 10.3389/fgene.2022.966939 (PMC9548531; doi:10.3389/fgene.2022.966939)

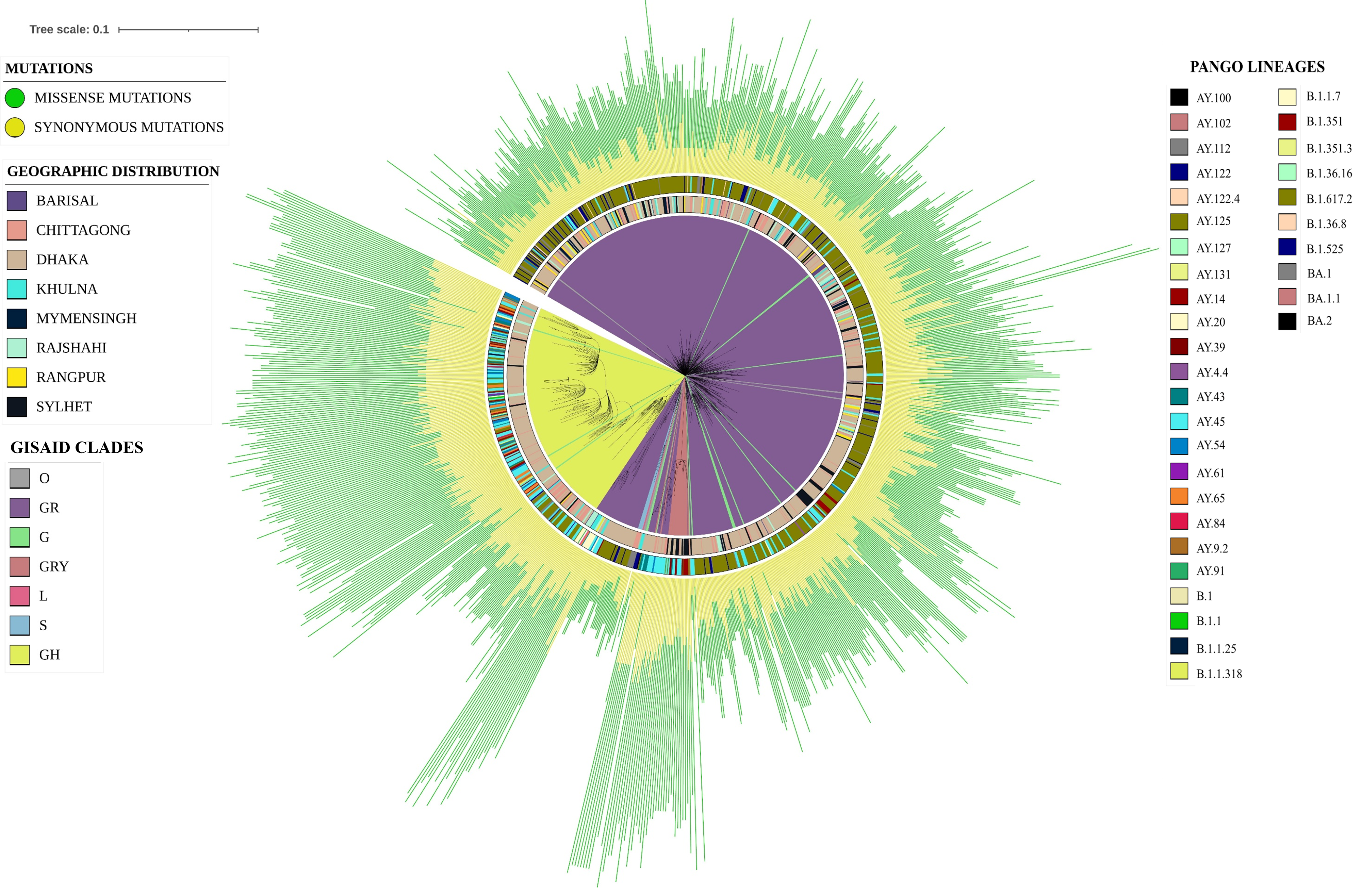

Supplement: Supplementary file 2 [file Image1.TIFF]

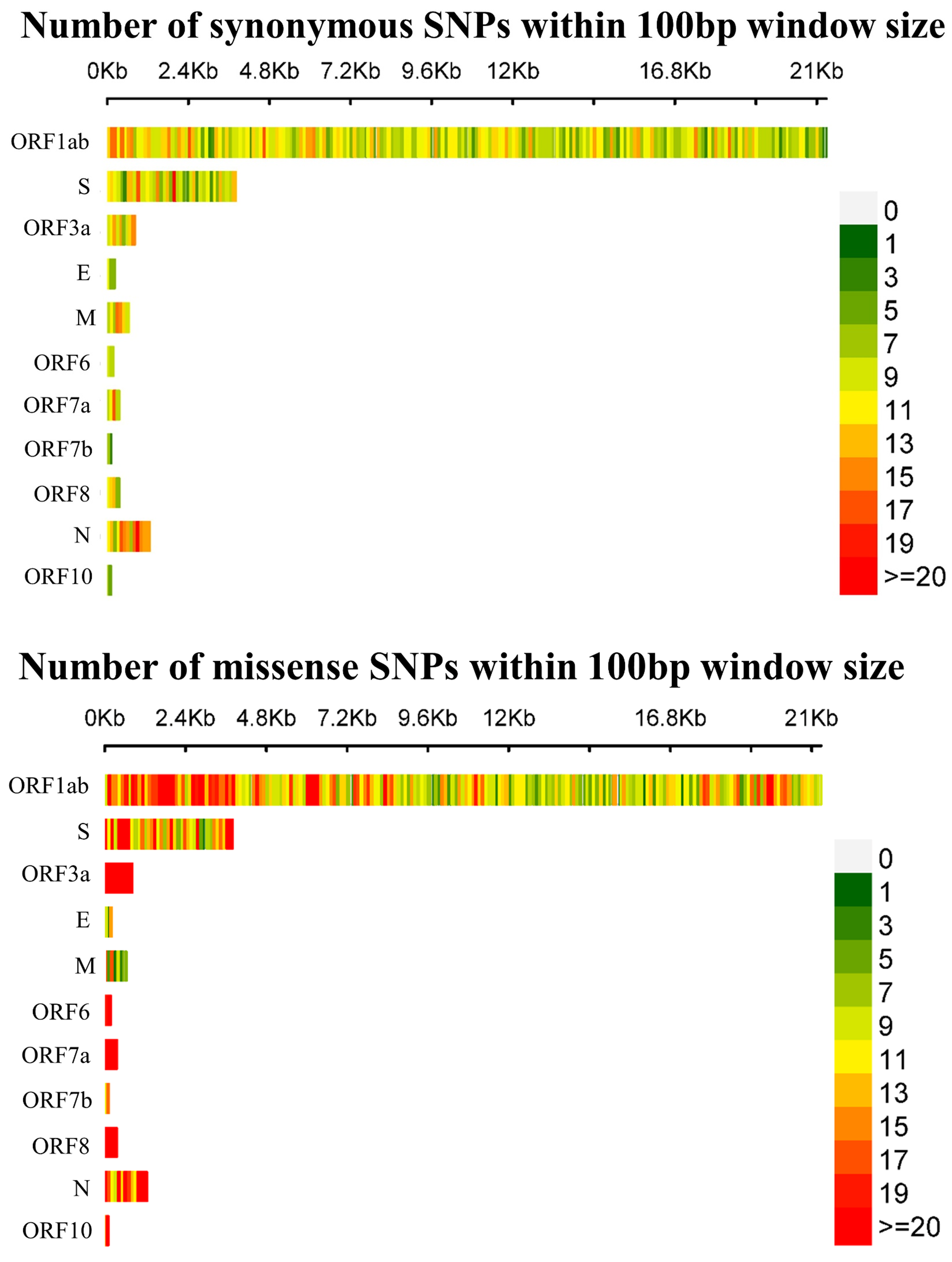

Supplement: Supplementary file 4 [file Image2.TIF]
